# Horticultural therapy for general health in the older adults: A systematic review and meta-analysis

**DOI:** 10.1371/journal.pone.0263598

**Published:** 2022-02-10

**Authors:** Zhijie Wang, Yu Zhang, Shanshan Lu, Linlin Tan, Wei Guo, Mark Lown, Xiaoyang Hu, Jianping Liu

**Affiliations:** 1 Department of Oncology, Shanxi Province Hospital of Traditional Chinese Medicine, Taiyuan, Shanxi, China; 2 Department of Tuina, The First Affiliated Hospital of Anhui University of Chinese Medicine, Hefei, An’hui, China; 3 Beijing Laboratory of Urban and Rural Ecological Environment, Beijing Floriculture Engineering Technology Research Center, Beijing Botanical Garden, Beijing, China; 4 The First Affiliated Hospital of Guangzhou University of Chinese Medicine, Guangzhou, Guangdong, China; 5 Primary Care, Population Sciences, and Medical Education, Faculty of Medicine, University of Southampton, Southampton, United Kingdom; 6 Center for Evidence-based Chinese Medicine, Beijing University of Chinese Medicine, Beijing, China; The University of Mississippi Medical Center, UNITED STATES

## Abstract

**Background:**

With a pronounced and historically unprecedented tendency of population ageing, research on ageing and related disorders has been increasingly brought into focus. Horticultural therapy (HT), as an important role of social prescribing, has been an integrative for decades. This systematic review and meta-analysis aimed to evaluate HT for general health in older adults.

**Methods:**

Electronic databases including PubMed, Web of Science, ScienceDirect, the Cochrane Library, MEDLINE (Ovid), EMBASE (Ovid), CINAHL (EBSCO), China National Knowledge Infrastructure (CNKI), and the Wanfang database, grey literature databases and clinical trials registers were searched from inception to March 2021. Randomized controlled trials (RCTs), quasi-RCTs (QRCTs) and the cohort studies about HT for adults aged over 60 were included in this study. Outcome measures were physical function, quality of life, BMI, mood tested by self-reported questionnaire and the expression of the immune cells. The study was registered under PROSPERO (CRD42019146184).

**Results:**

Totally, fifteen studies (thirteen RCTs and two cohort studies) involving 1046 older participants were included. Meta-analysis showed that HT resulted in better quality of life (MD 2.09, 95% CI [1.33, 2.85], P<0. 01) and physical function (SMD 0.82, 95% [0.36, 1.29], P<0.01) compared with no-gardener; the similar findings showed in BMI (SMD -0.30, 95% [-0.57, -0.04], P = 0.02) and mood tested by self-reported questionnaire (SMD 2.80, 95% CI [1.82, 3.79], P<0. 01). And HT might be conducive on blood pressure and immunity, while all the evidence were moderate-quality judged by GRADE.

**Conclusions:**

HT may improve physical function and quality of life in older adults, reduce BMI and enhance positive mood. A suitable duration of HT may be between 60 to 120 minutes per week lasting 1.5 to 12 months. However, it remains unclear as to what constitutes an optimal recommendation.

## 1 Background

With a pronounced and historically unprecedented tendency of population ageing, research on ageing and related disorders has been increasingly brought into focus. At the end of 2017, 6.7% (800 million) of the global population were aged 60 years or older, and in the next four decades, this is projected to rise to 22% (2 billion) [1]. This situation is more concerning in rural regions with lower population densities due to a lack of economic and educational resources that causes more young people to move to developed regions, leaving behind increasingly isolated elders [2]. Syndromes such as frailty, sarcopenia and illnesses including Parkinson’s, dementia, type 2 diabetes, and cardiovascular disorders are more common with increasing age, especially for those who live alone [3, 4]. Lifestyle factors such as poor nutrition and lack of physical activity are increasingly recognized to impact on older adults’ ability to maintain their independence [5]. Multi-morbidity is more common with increasing age often leading to the introduction of multiple medications that can in turn raise the risk of harm due to adverse reactions. Population ageing also brings serious concerns about economic consequences including the cost of general health management [6, 7]. In view of the rapid pace of population ageing and the heavy economic burden, some opinions suggest reducing the emphasis of most health systems on the provision of expensive treatment and care [8]. There is a great priority to improve the evidence for economical and cost-effective interventions to promote healthy ageing, reduce the proportion of elderly people falling below the disability threshold and contribute to the sustainability of health and social care systems [9].

To lower the cost of healthcare, social prescribing (SP) involving non-clinical services such as gardening, arts activities, and healthy eating advice has been an important aspect [10], and SP also helps patients overcome some social and behavioral determinants of poor health [11]. A growing body of evidence has proved that social prescribing could reduce pressure on the National Health Service (NHS) in the UK by offering people more appropriate services and groups [12]. Research reported that where participants were supported by SP, the consultations of general practitioner (GP) reduced by 28% and attendances at accident and emergency services by 24% on average [13]. More and more voices have been developed to support SP, while evaluations should be done on participant health outcomes and also challenges of setting up such a service [14].

Horticultural therapy (HT), also commonly referred to as therapeutic horticulture is defined as the participation of a person in gardening-related activities with help of trained therapists. HT is a subgroup of occupational therapy [15] and the aim of HT is to achieve a specific treatment goal such as to improve the symptoms of posttraumatic stress disorder or depression [16, 17]. At present, it is widely accepted that HT is an open process of caring for garden activities such as growing fruits, vegetables and/or flowers [18, 19], and a HT team always includes trained therapist, healthcare providers, patients or ordinary people to work together, which could bring more communications between the older.

HT has been widely accepted as a complementary and alternative medicine for decades, and may work in a number of ways. Therapeutic gardens with safety and physical comfort designing have appeared in many hospitals, nursing houses and rehabilitation centers which are nature-orientated space of sanctuary and facilitate contact with nature [20]. This type of gardens may help reduce stress and depression levels in patients suffering from mental disorders, which may lead to improvements in thinking and more positive emotions [21]. Community gardens, home-based gardeners and potted plants have been shown not only to reduce the cost of foods, but also improve mental health, cardiovascular health and improve overall quality of life status [22–26].

The staggering costs of aged-relative diseases mean prevention and management of conditions in older people is important. Many localities are now and considering implementing HT because it is a holistic approach including physical, cognitive, psycho-emotional, and social prescribing [27], but it is unclear that whether HT help to promote health of the older. A systematic review is required to identify the clinical trials in HT area and summarize the evidence for healthcare professionals, researchers, policy makers and others with an interest in HT for older people.

## 2 Methods

This systematic review was reported following the Preferred Reporting Item for Systematic Reviews and Meta-Analysis (PRISMA) [28] and registered with the PROPESRO CRD42019146184 [29].

### 2.1 Data sources and search terms

Electronic databases, grey literature databases and clinical trials registers were researched from inception to March 2021. Electronic databases included PubMed, Web of Science, ScienceDirect, the Cochrane Library, MEDLINE (Ovid), EMBASE (Ovid), CINAHL (EBSCO), China National Knowledge Infrastructure (CNKI), and the Wanfang database; grey literature databases included OpenGrey (www.OpenGery.eu); and clinical trials registers included Clinicaltrials.gov (www.ClinicalTrials.gov). There were no restriction on publication language and regions. The searching strategy followed the guidance of the Cochrane Review Handbook, and a detailed search strategy is available in the S1 Appendix.

### 2.2 Criteria for considering studies

#### 2.2.1 Types of studies

All clinical studies including randomized controlled trials (RCTs), quasi RCTs, cohort studies, cross-sectional studies, and case-control studies which observing HT for older adults was included in this systematic review. No language restrictions were imposed.

#### 2.2.2 Types of participants

Participants were age ≥ 60 years irrespective of gender, ethnicity, or basic health conditions.

#### 2.2.3 Types of interventions

Any form of HT involving therapeutic gardens, allotment gardening, and home gardening, compared with traditional activities, waiting list, non-gardener, not using any kind of HT, or placebo.

#### 2.2.4 Types of outcome measures

The main outcomes were quality of life and physical function. Other outcomes of interest included body mass index (BMI), mood related patient reported outcomes, blood pressure and immunity of participants.

Quality of life was measured by the Short Form-36 (SF-36, higher scores indicates higher quality), Activities of Daily Living Scale (ADL, higher scores indicates lower quality), Barthel Index (higher scores indicates higher quality), Quality of Life Index (higher scores indicates higher quality) or other validated scales; physical function, measured by balance test, 2-min step test, chair stand test, arm curl test, chair sit-and-reach test (higher score indicates better function of all involved test), myodynamia, blood pressure or other validated tests. Body mass index (BMI) was measured as weight divided by height squared (kg/m^2^) of participants; mood was tested by self-reported questionnaire; immunity was tested by the expression of the immune cells.

### 2.3 Data extraction and management

References were downloaded from databases, journals or entered manually into Endnote for de-duplicating. Two authors extracted the following data from included studies and put the data into Review Manager Version 5.3 (The Nordic Cochrane Centre, The Cochrane Collaboration, 2014) independently. Study characteristics extraction included: study ID, conducted location, basic health condition of participants, sample size, age, interventions in all study arms, outcomes, and follow-ups (if necessary).

### 2.4 Risk of bias and reporting quality of included studies

Two authors (ZY and GW) assessed the reporting quality and methodology quality using Cochrane Collaboration risk of bias tool independently. Low, unclear, or high risk of bias was judged in seven domains for every included study using the tool [30]. Disagreements were discussed and resolved, and a third author (WZJ) gave advice in case of no consensus. The evidence level of the included studies was assessed by Grades of Recommendations Assessment, Development and Evaluation (GRADE) [31]. Results including quality of life, physical function, and mood assessed by self-reported questionnaire of this study are reported in the summary of findings tables. The level of evidence was assessed as high, moderate, low or very low.

### 2.5 Measure of treatment effect

The meta-analysis was analyzed using Review Manager Version 5.3 (The Nordic Cochrane Centre, The Cochrane Collaboration, 2014). For continuous outcomes, scores at the end of treatment rather than change from baseline were extracted. For continuous data, because of the anticipated variability in the populations and the medical conditions of included studies, a generic inverse variance random effects model was used to pool the mean differences (MD) with 95% confidence interval (CI) to incorporate heterogeneity, and if different scales were used to measure the same outcome, standardized mean difference (SMD) was used [30]. Risk ratio (RR) with corresponding 95% CI for dichotomous outcomes was presented as point estimates.

### 2.6 Dealing with missing data

Study authors were planned to be contacted if data was missing or incomplete where possible. When standard deviation was not reported with means, it was calculated from the information reported such as CIs, *P*-values, or *F*-values. The number of participants whose data was available at baseline and at the last follow-up, and the rate of loss to follow-up were recorded.

### 2.7 Assessment of heterogeneity

Between-study heterogeneity was assessed using the *I*^2^ statistics test which describes the percentage of variation across studies that was due to heterogeneity rather than chance. Rules of thumb for interpretation of this statistic suggest that *I*^2^ > 30% represented moderate heterogeneity, *I*^2^ > 50% represented substantial heterogeneity, and *I*^2^ > 75% represented considerable heterogeneity [30]. If *I*^2^ values > 50%, potential sources of heterogeneity were further investigated in a sensitivity analysis and taken into account when interpreting the findings and a random-effects model was conducted to obtain a pooled estimate of effect; otherwise a fixed-effects model was used. A narrative description was reported when a meta-analysis could not be performed due to small numbers of included studies.

### 2.8 Subgroup analysis

If sufficient data was available, subgroup analyses was performed to compare the effect estimate between included studies. Subgroup analyses were conducted for different comparisons, and different outcome measures. Data from each subgroup was synthesized and evaluated independently.

### 2.9 Assessment of reporting bias

Funnel plot tests for asymmetry were conducted to investigate potential reporting bias if there were sufficient studies under a single meta-analysis [32].

## 3 Results

**Fig 1:**
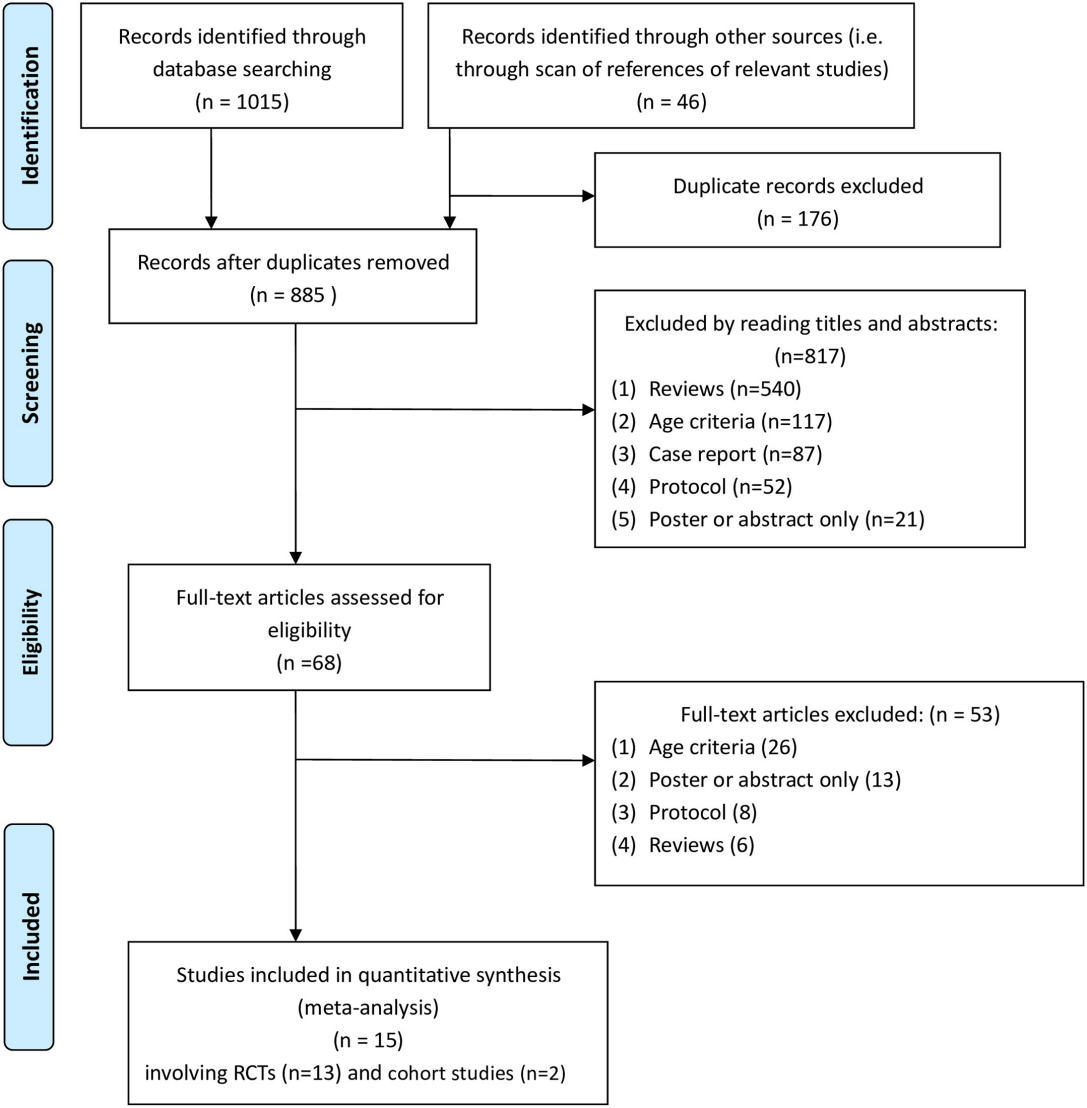
Literature screening process and results.

A total 1,061 potentially relevant studies were identified. After the removal of duplicates, 885 titles and abstracts on HT for older adults were screened independently (WZJ, ZY, GW); after the removal of scanning titles and abstracts, 68 studies were scanned by applying inclusion and exclusion criteria, and a total fifteen studies [33–47] remained eligible (WZJ, ZY, GW), thirteen of them were RCTs [33–45] and two of them were cohort studies [46, 47] (Fig 1).

### 3.1 Study characteristics

Study characteristics of the included RCTs are presented in Table 1. 974 participants (13–180 per trial) were included from the thirteen trials, which were published between 2010 and 2021. Eight of them were conducted in China [34, 35, 37–40, 42–44], two in USA [33, 36], one in Singapore [45] and one in South Korea [41], and one conducted in cross-country including China, India and others Asia countries [39]. Nine of the included trials were published in English, and four in Chinese. Four trials recruited healthy older adults [35, 37, 39, 43], two recruited participants suffering from dementia [33, 34], one recruited cancer survivors [36], one recruited patients with mild Alzheimer’s disease [38], one focused on older people with depression [44], one focused on stroke [42], one focused on frail and prefrail nursing home residents [40], one study recruited patients with mental health problems including cognitive impairment, depressive disorder and anxiety disorder [41], and one included all types of older people including healthy or with high blood pressure, diabetes, heart attack, arthritis, and depression [45]. Eleven included trials [33–35, 37, 39–45] reported the duration of HT ranging from 30 min to 120 min for each therapy lasting 1.5 to 12 months. All included trials reported that participants were under supervision by training staff during the entire study period to ensure safety, establish strong interactive relationships and build self-efficacy for the older participants.

**Table 1:** Study characteristics of included RCTs on horticultural therapy for the older.

study ID (author & year)	location	condition of participants	observation group			control group			outcomes	follow-up
			sample size	age (year)	intervention	sample size	age (year)	intervention		
Shannon 2010 [33]	USA	dementia	75	81.34±7.17	horticultural therapy-based activities (30 min×2 per week)	54	78.36±8.92	traditional activities	mood score	1.5 months
Luk 2011 [34]	Hong Kong, China	dementia	7	84.9±8.3	allotment gardening (30min×2 per week)	6	84.9±8.3	NR	mood score	1.5 months
Yang 2016 [35]	Mainland, China	healthy people	90	>65	allotment gardening (40min×3 per week)	90	>65	nothing	BMI, physical function, mood score	8 months
Wahnefried 2018 [36]	USA	cancer survivors	24	70.1±8.1	allotment gardening	22	69.7±8.5	waiting list	BMI, physical function, quality of life, mood score	12 months
Yao 2017 [37]	Taiwan, China	healthy people	41	77.41±8.56	allotment gardening (60min×1 per week)	44	82.55±7.50	nothing/ waiting list	quality of life, mood score	2 months
Chen 2018 [38]	Mainland, China	mild Alzheimer’s disease	35	73.41±9.81	therapeutic gardens (2 times per week)	33	76.13±11.18	nothing	quality of life	12 months
Kheng 2018 [39]	Asia (China, India, others)	healthy people	29	67.21±4.52	horticultural therapy (60min×1 per week)	30	67.00±4.18	waiting list	mood score	6 months
Lai 2018 [40]	Hong Kong, China	frail and prefrail nursing home residents	46	85.54±6.72	horticultural therapy (60-minute program for 8 weeks)	50	83.74±7.66	traditional activities	mood score	2 months
Han 2018 [41]	South Korea	mental health problems	14	80.1±2.9	allotment gardening (90min×1 per week)	14	77.4±5.9	nothing	physical function	2 months
Yan 2019 [42]	Mainland, China	stroke	20	61.82±3.04	horticultural therapy (30min/d, for 14 days)	20	63.05±2.74	acupuncture	physical function	NR
Wang 2020 [43]	Mainland, China	healthy people	28	83.50±2.35	allotment gardening (90min×1 per week)	30	83.20±1.97	nothing	physical function, mood score	2 months
Wangz 2020 [44]	Mainland, China	depression	63	67.92±2.58	horticultural therapy (60-120min/d) + traditional activities	63	68.57±2.73	traditional activities	mood score	NR
Wong 2021 [45]	Singapore	all people	22	67.21±4.52	horticultural therapy (15 hourly per week in the first 3 months, 15 hourly per month in the remaining 3 months)	24	67.00±4.18	waiting list	expression of immune cell	6 months

NR: not reported; nothing: participants did not change their life styles and lived as usual at home or nursing house; traditional activities: participants did the same activities as usual.

Study characteristics of the included cohort studies are presented in Table 2. Seventy-two participants (34–38 per trial) were included from the two studies, which were both published in 2020 and conducted in mainland China. One study recruited healthy older adults [46] and another were dementia [47]. One observed potting for 25 min per day compared to flower arranging for 25 min per day in effect on the change of mood score [46], and one observed HT 30 min per time lasting four times with doing nothing in effect on the physical function [47].

**Table 2:** Study characteristics of included cohort studies on horticultural therapy for the older.

study ID (author & year)	location	condition of participants	arm 1			arm 2			outcomes
			sample size	age (year)	intervention	sample size	age (year)	intervention	
Huang 2020 [46]	Mainland, China	healthy people	19	65–99	Potting (25min)	19	65–99	flower arranging (25min)	mood score
Li 2020 [47]	Mainland, China	dementia	17	80.69±8.23	horticultural therapy (30min per time, for 4 times)	17	81.87±9.05	nothing	physical function

Nothing: participants did not change their life styles and lived as usual at home or nursing house.

### 3.2 Risk of bias in included RCTs

**Fig 2:**
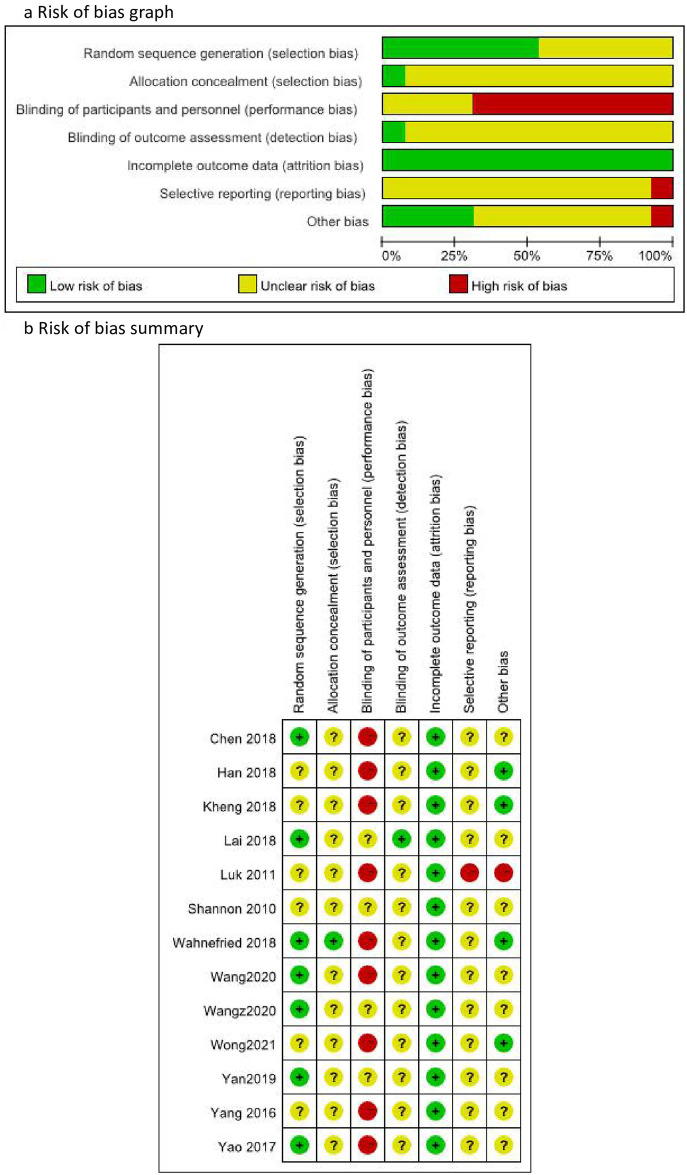
Risk of bias of RCTs on horticultural therapy.

Methodological quality of the included RCTs was poor (Fig 2). Seven [36–38, 40, 42–44], of the included studies reported using random sequence generation methods using random number blocks, which was assessed as having a low risk of bias; others did not report the randomization method, and hence the risk of bias was unclear. Low risk of bias of allocation concealment was assessed for one trial [36] by using sealed envelopes, and the others had no information on allocation concealment. All included trials did not report the information on blinding of participants, while the participants in eight of them [35–39, 41, 43, 45] did nothing or waiting list which they should be aware of which group was divided, so high risk of bias was assessed. One trial [40] reported the information on blinding of outcome assessment so low risk was assessed, while others were lack of information which was assessed as unclear risk of bias. All studies reported reasons for drop-out of participants which indicated low risk of attrition bias. It was difficult to assess reporting bias in all included studies because of none of them reported a protocol, which indicated unclear risk of bias. Four trials [36, 39, 41, 45] reported detailed information on ethics, funding, authors’ details and other related information, which indicated low risk of other biases, while other trials reported limited information (assessed as unclear risk of bias). One trial [34] did not report the intervention in control group, so high risk of bias was assessed in selective reporting and other bias.

### 3.3 Effect estimation

#### 3.3.1 Quality of life

Three studies reported quality of life as an outcome, using SF-36 [36], Barthel Index [37], and quality of life index [38]. It showed that HT could improve the quality of old people with mild Alzheimer’s disease when measured by quality of life index (MD 2.09, 95% CI [1.33, 2.85], P<0.01) by SF-36, while had no promoting effect on cancer survivors (MD 2.02, 95% CI [-0.56, 4.60], P = 0.12) by Barthel Index or healthy old people (MD 5.27, 95% CI [-6.08, 16.62], P = 0.36) by quality of life index.

#### 3.3.2 Physical function

**Fig 3:**
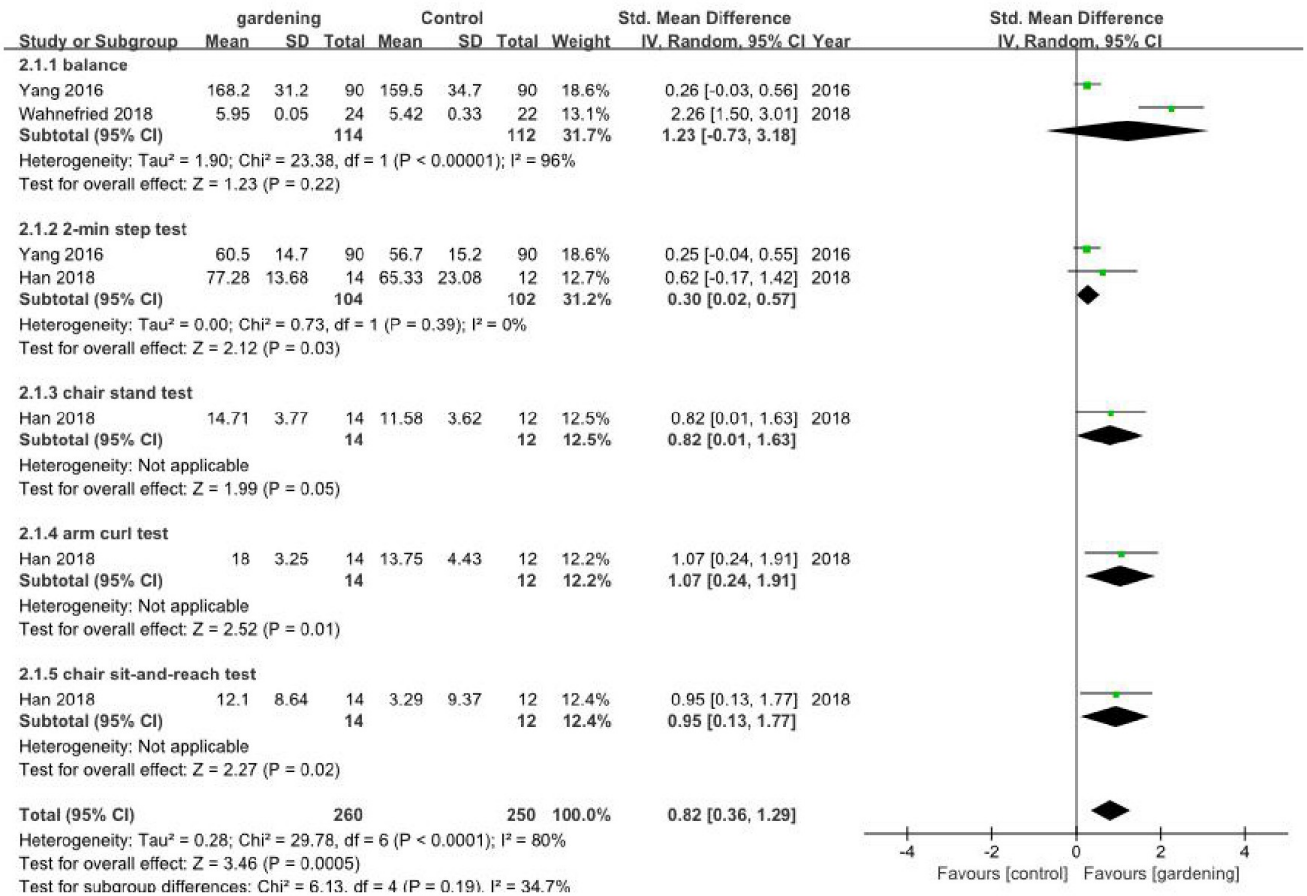
Meta-analysis of physical function of the included RCTs.

Three included studies reported physical function involving five tests as an outcome. Two studies [35, 36] tested balance, two studies [35, 41] carried out a 2-min step test, one [41] performed a chair stand test, arm curl test and chair sit-and-reach test. The results showed that gardening had larger positive effect on physical function than control group (SMD 0.82, 95% [0.36, 1.29]; *I*^2^ = 80%, P<0.01). Because of high heterogeneity, subgroup analysis was performed according to different tests; balance test-no different between gardening and non-gardening (SMD 1.23, 95% [-0.73, 3.18]; *I*^2^ = 96%, P<0.01); 2-min step test-gardening showed better effect than control (SMD 0.30, 95% [0.02, 0.57]; *I*^2^ = 0%, P = 0.03); chair stand test-gardening did not showed significant effect than control (SMD 0.82, 95% [0.01, 1.63], P = 0.05); arm curl test-gardening showed better effect than control (SMD 1.07, 95% [0.24, 1.91], P = 0.01); chair sit-and-reach test-gardening showed better effect than control (SMD 0.95, 95% [0.13, 1.77], P = 0.02) (Fig 3).

One study [42] evaluated the function of the wrist including myodynamia (measured by the peak torque, higher value means better) and activity (higher value means better) of the stroke old patients. The results showed that HT plus acupuncture had better effect on myodynamia (11.36±2.99 vs. 10.36±2.12) (N·m) and activity (150.36±17.12 vs. 135.36±15.12) (°) compared to acupuncture only.

One trial [43] evaluated the blood pressure of the older, and the results showed that compared to doing nothing, HT might maintain the stable blood pressure, especially in systolic pressure (136.54±3.38/71.86±3.71 vs. 152.40±3.64/65.90±3.33) (mmHg).

#### 3.3.3 BMI

Two studies [35, 36] evaluated BMI, and the findings showing that HT could reduce BMI of older adults compared with participants in control groups (SMD -0.30, 95% [-0.57, -0.04]; *I*^2^ = 0%, P = 0.02).

#### 3.3.4 Mood was tested by self-reported questionnaire

**Fig 4:**
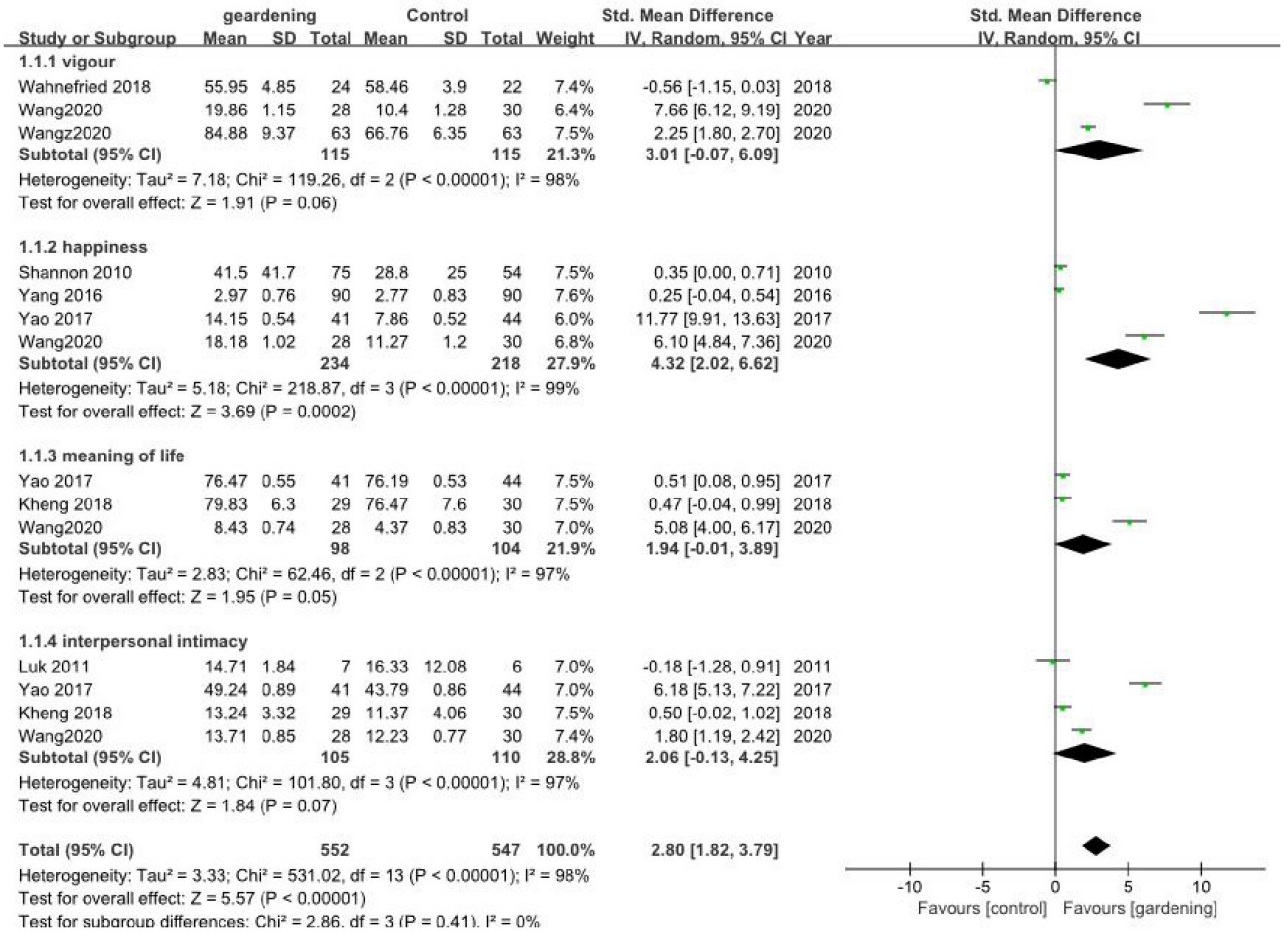
Meta-analysis for mood tests of the included RCTs.

Eight included trials performed mood tests as outcomes. Three [36, 43, 44] measured vigor, four [33, 35, 37, 43] tested happiness, three [37, 39, 43] tested meaning of life, and four [34, 37, 39, 43] tested interpersonal intimacy. The result showed that gardening could improve positive effect of mood in older adults compared to the control group (SMD 2.80, 95% CI [1.82, 3.79]; *I*^2^ = 98%, P<0.01). As there was high heterogeneity, subgroup analysis was performed according to different tests. Only for happiness, HT showed a positive effect (SMD 4.32, 95% CI [2.02, 6.62]; *I*^2^ = 99%, P = 0.0002), while for other three emotions, there were no significant differences between HT and the control groups: for vigor (SMD 3.01, 95% CI [-0.07, 6.09]; *I*^2^ = 98%, P = 0.06), for meaning of life (SMD 1.94, 95% CI [-0.01, 3.89]; *I*^2^ = 97%, P = 0.05), or for interpersonal intimacy (SMD 2.06, 95% CI [-0.13, 4.25]; *I*^2^ = 97%, P = 0.07) (Fig 4).

### 3.4 The expression of the immune cells

One trial [45] reported the relationship between HT and immune function, and the results showed that HT was associated with a reduction in the proportion of CTLA4+ TEMRAs and CTLA+CD8+ TEMRAs, and increasing the absolute numbers of naive CD8T cells when compared to waiting list group.

### 3.5 Evidence level based on GRADE

Based on the results from GRADE, moderate-quality evidence of HT may increase physical function, quality of life, mood test for older adults, and BMI in this review. The main cause of the down-grading was the interventions in the control groups were doing nothing or waiting list, so the participants should know which group they were in (S2 Appendix).

### 3.6 Results from cohort studies

Totally two cohort studies were included in this review, one [46] conducted allotment gardening as the interventions, and the results showed that it might lower the high blood pressure (152.38±24.71/89.19±26.32 vs. 132.06±16.07/73.65±12.84) (mmHg) after four HT treatments. Another study analyzed the effect of the horticultural activities on the emotions of the older, and the results showed that flower arrangement and succulent plants potting both could improve the emotions losing interest in things, but the former had better effect than the latter (*F* (1, 31) = 11.717, *p* = 0.002, η^2^_p_ = 0.274).

## 4 Discussions

This systematic review focused on evaluating HT for older adults. Though limitations existed, this study still proved some promising evidence that HT could be a recommendable intervention for older adults to improve quality of life and physical functions.

### 4.1 Summary of main results

Fifteen studies were eligible for this systematic review, and the results showed that HT may improve physical function and quality of life of older adults with mild Alzheimer’s disease, reduce BMI and improve the mood of happiness, improve the immunity. This may have more opportunities to communicate with others and the natural world through gardening as all the included trials conducted as a collective therapy under supervision by training staff. The suitable duration for HT may be 60 min to 120 min per week for each therapy lasting from 1.5 months to 12 months, while it remains unclear as to what constitutes an optimal recommendation. Weight of older people is associated with physical function, and overweight may lead to many health problems such as type 2 diabetes, cardiovascular disease, hypertension, and osteoarthritis of the knees. The most commonly used measure for classifying overweight is BMI, and in adults a desirable BMI is between 18.5 and 25 and overweight between 25 and 30. From the result of meta-analysis, HT may be an effective way to reduce BMI in older adults. The similar result showed in testing of mood, positive mood may be significant in older adults, helping individuals to satisfy with themselves and others, living better in the final stage of their lives [41]. Evidence from the included studies was certainly relevant to the main question of this systematic review, but it was not sufficient to address all of the problems because of no enough number of relevant RCTs. The short duration of intervention and follow-up in those included studies might influence the directness of evidence, considering the chronic nature of conditions of the older. Overall, the evidence in the included studies was low assessed by GRADE, as most of the included trials (n = 7) were assessed as high risk of performance bias because of no information on blinding; one trial [37] did not report detail information on what intervention used in the control group, which led to high risk bias of selective and other bias. While high heterogeneity in some results may be because of small numbers of included studies, different interventions in the control group, difference duration of treatment or different scales used for the same test. In addition, race, gender, age, co-morbidity, education level and basic physical condition of the older could also affect the heterogeneity of the results.

### 4.2 Comparison with other studies or reviews

The findings in this systematic review suggest that HT seems to be a beneficial intervention for general health of older people. Some similar findings have reported in previous research. A survey reported that general adults aged 16–99 years with or without heart conditions may benefit from gardening in respect of ability to concentrate, feeling capable of making decisions, and self-worth [42]. A evidence summary reported in China proposed that HT should be an intervention for older adults with dementia because it may improve physical function and emotions, while shortcomings such as lacking gardens in many communities and nursing homes in China was highlighted [43]. A controlled cohort study with seventy-nine inpatients in Switzerland showed that HT could improve the physical and mental health evaluated by the Medical Outcome Study Short Form-36, reduce anxiety and depression levels using the Hospital Anxiety and Depression Scale, and reduce the chronic musculoskeletal pain [44]. A cohort study with 11-year follow-up showed that daily home-based gardening was associated with a higher survival rate in old adults with mobility limitations in Taiwan, China [45]. Gardening may reduce diabetes through increasing physical activity and the intake of nutritious food such as fresh vegetables and fruits [46]. Community gardens may provide health benefits including improved access to food and increased physical function in South-East Toronto [22]. A systematic review showed a positive association between gardening and obesity but it did not provide evidence on mechanisms [47]. However, some case-report studies reported that gardening may also be associated with health risks: a case report showed that home gardening may be a risk factor for contact dermatitis to Alstroemeria [48]; Aspergillus spores are often found on decaying plant matter, which may cause a lethal allergic symptom of older adults with chronic diseases. A case reported one person died after dispersing rotting tree and plant mulch in the garden and the diagnosis of aspergillosis was confirmed when analyzed two serum samples of this patient by the Mycology Reference Centre, Leeds, UK [49]. Although lethal cases are rare, the health state of participant especially who is allergic constitution should be monitored at all times during the whole treatment. So future public health programs promoting HT should be encouraged, but need some training and be under supervision. Those findings are increasingly recognized that contact horticultural regularly could promote the health.

### 4.3 Strengths

This systematic review was a comprehensive evaluation of HT for quality of life in older adults, and the evidence from not only RCTs, but also cohort studies. Though previous studies reported HT was benefit for quality of life, there were few studies focused on physical function, mood or immune function. Predefined subgroup analysis and CRADE for the evidence level of outcomes were conducted in this systematic review.

### 4.4 Limitations

Firstly, the most important limitation in this study was that different scales were employed for same outcome, which introduced high heterogeneity. Secondly, small number of trials identified with small number of participants is another important limitation in this review; thirdly, there is a gap from the search data to the publication data, so during this time a number of eligible studies may be conducted and published whose impact on the findings of this systematic review is uncertain. And the included studies mainly carried out in Asian countries, although the search for studies was thorough, potential biases may still exist. Finally, no included trials evaluated cost effectiveness. SP has been developed to a health pathway combines clinical and non-clinical activities together. 1,000 new social prescribing link works will be recruited by 2020/2021 in the UK [14], while the cost by SP should be taken into account.

### 4.5 Future direction

On the whole quality of the reporting of trials in the meta-analysis was low, more rigorous RCTs with precise methodological design and complete reporting on HT for health of older adults would be promoted. There was insufficient evidence of gardening regimen in the included trials, so it should be performed detailed in future research, and it is important for future research to have standardized recommendations and treatment duration for conducting HT and reporting of related outcomes, better reporting of all the parameters of HT and the accuracy of measurements. And another key area for future studies is improving understanding of the mechanisms through how horticultural can improve health for the older.

## 5 Conclusions

HT may improve physical function and quality of life in older adults, reduce BMI and improve mood. A suitable duration for HT may be between 30 min to 90 min for each therapy lasting from 1.5 months to 12 months. However the current findings in this study did not provide strong evidence of HT for health outcomes of older adults due to low quality and small sample size.

## Supporting information

S1 Appendix Search strategy. (PDF)

S2 Appendix Evidence level based on GRADE. (PDF)

S1 Checklist Prisma 2020 checklist. (DOCX)
