# Supplementary material for: Horticultural therapy for general health in the older adults: A systematic review and meta-analysis
Source: PLoS One. 2022 Feb 10;17(2):e0263598. doi: 10.1371/journal.pone.0263598 (PMC8830630; doi:10.1371/journal.pone.0263598)
Supplement: S1 Appendix — (PDF) [file pone.0263598.s001.pdf]

Appendix 1 Search strategy

PubMed

(horticultural therapy[Title/Abstract] OR gardening[Title/Abstract] OR therapeutic garden[Title/Abstract] OR horticulture[Title/Abstract] OR garden[Title/Abstract]) AND ("aged"[MeSH Terms] OR "aged"[All Fields] OR "elderly"[All Fields] OR "older"[All Fields]) AND (randomized controlled trial[Title/Abstract] OR clinical study[Title/Abstract] OR group[Title/Abstract])

Embase (Ovid)

(horticultural* therapy) OR (gardening) OR (therapeutic* garden) OR (horticulture) AND (aging*) OR (aged*) OR (elderly) AND (randomized controlled trial) OR (controlled study) OR (clinical trial)

Medline (Ovid)

(“horticultural therapy*”) OR (gardening) OR (“therapeutic horticultural*”) OR (therapeutic garden) OR (garden) AND (aged*) OR (elderly) OR (old) AND (randomized controlled trial) OR (clinical trial) OR (clinical trial, phase Ⅰ) OR (clinical trial, phase Ⅱ) OR (clinical trial, phaseⅢ) OR (clinical trial, phase Ⅳ) OR (clinical trial, protocol)
