# Supplementary material for: Horticultural therapy for general health in the older adults: A systematic review and meta-analysis
Source: PLoS One. 2022 Feb 10;17(2):e0263598. doi: 10.1371/journal.pone.0263598 (PMC8830630; doi:10.1371/journal.pone.0263598)
Supplement: S2 Appendix — (PDF) [file pone.0263598.s002.pdf]

Appendix 2 Evidence level based on GRADE

HT for Quality of life of elder adults

Patient or population: the older with HT

Settings: RCTs

Intervention: horticultural therapy (HT)

Outcomes	Illustrative comparative risks* (95% CI)		Relative effect (95% CI)	No of Participants (studies)	Quality of the evidence (GRADE)	Comments
	Assumed risk	Corresponding risk				
	**Control**	**HT**				
**Quality of life**<br>SF-36, Barthel Index, quality of life index<br>Follow-up: 2-12 months		The mean quality of life in the intervention groups was<br>**0.59 standard deviations higher**<br>(0.3 to 0.88 higher)		199<br>(3 studies)	⊕⊕⊕⊖<br>**moderate**[1]	
**physical function**<br>balance, a 2-min step test, a chair stand test, arm curl test, chair sit-and-reach test<br>Follow-up: 2-12 months		The mean physical function in the intervention groups was<br>**0.82 standard deviations higher**<br>(0.36 to 1.29 higher)		254<br>(3 studies)	⊕⊕⊕⊖<br>**moderate**[1]	
**BMI**<br>weight (kg)/high2 (m2)<br>Follow-up: 8-12 months		The mean bmi in the intervention groups was<br>**0.25 lower**<br>(0.48 to 0.02 lower)		226<br>(2 studies)	⊕⊕⊕⊖<br>**moderate**[1,2]	
**mood test**<br>vigor, happiness, meaning of life, interpersonal intimacy<br>Follow-up: 1.5-12 months		The mean mood test in the intervention groups was<br>**2.80 standard deviations higher**<br>(1.82 to 3.79 higher)		696<br>(8 studies)	⊕⊕⊕⊖<br>**moderate**[3]	

*The basis for the **assumed risk** (e.g. the median control group risk across studies) is provided in footnotes. The **corresponding risk** (and its 95% confidence interval) is based on the assumed risk in the comparison group and the **relative effect** of the intervention (and its 95% CI).

CI: Confidence interval;

GRADE Working Group grades of evidence

High quality: Further research is very unlikely to change our confidence in the estimate of effect.

Moderate quality: Further research is likely to have an important impact on our confidence in the estimate of effect and may change the estimate.

Low quality: Further research is very likely to have an important impact on our confidence in the estimate of effect and is likely to change the estimate.

Very low quality: We are very uncertain about the estimate.

[1] Not reported the blinding, and the interventions in the control group were nothing changed as usual, so the participants would know which group they were in.

[2] One (Wahnefried 2018) of the included trials was assessed as low risk of bias of selection, attrition, and other bias.

[3] One (Luk 2011) of the included trials did not report details of intervention in control group, and five of the included trials were assessed performance bias as high risk.
